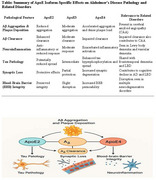# Elucidating the Isoform‐Specific Effects of Apolipoprotein E on Amyloid‐Beta Clearance and Neuroinflammation in Alzheimer’s Disease and Related Dementias

**DOI:** 10.1002/alz70861_108267

**Published:** 2025-12-23

**Authors:** Chanikarn Sonpee, Chanida Ruchisrisarod, Pasin Hemachudha, Thirawat Supharatpariyakorn, Watayuth Luechaipanit, Abhinbhen Wasontiwong Saraya, Poosanu Thanapornsangsuth

**Affiliations:** ^1^ Kingchulalongkorn Memorial Hospital, Pathumwan, Bangkok Thailand; ^2^ Chulalongkorn university, Pathumwan, Bangkok Thailand; ^3^ Thai Red Cross Emerging Infectious Diseases Health Science Centre, King Chulalongkorn Memorial Hospital, Bangkok Thailand; ^4^ Elderly Health Care Center, Queen Savang Vadhana Memorial Hospital, Sriracha, Chonburi Thailand; ^5^ Faculty of Medicine, Chulalongkorn University, Bangkok Thailand; ^6^ King Chulalongkorn Memorial Hospital, Bangkok Thailand; ^7^ Chula Neuroscience Centre, Bangkok, Bangkok Thailand; ^8^ King Chulalongkorn Memorial Hospital The Thai Red Cross Society, Bangkok Thailand; ^9^ Thai Red Cross Emerging Infectious Diseases Health Science Centre, World Health Organization Collaborating Centre for Research and Training on Viral Zoonoses, King Chulalongkorn Memorial Hospital, The Thai Red Cross Society, Bangkok Thailand; ^10^ Division of Neurology, Department of Medicine, Faculty of Medicine, Chulalongkorn University, Bangkok Thailand; ^11^ Chulalongkorn University, Bangkok Thailand; ^12^ Chula Neuroscience Center, King Chulalongkorn Memorial Hospital, The Thai Red Cross Society, Bangkok Thailand

## Abstract

**Background:**

Alzheimer’s disease (AD) is a progressive neurodegenerative disorder and the most common cause of dementia worldwide. Its pathogenesis is closely linked to the accumulation of amyloid‐beta (Aβ) peptides and the influence of apolipoprotein E (ApoE) genotypes, particularly the ε4 allele. Understanding the roles of Aβ and ApoE in AD is critical for identifying early biomarkers and therapeutic targets, especially given the overlap in pathological mechanisms seen in related neurodegenerative disorders.

**Method:**

A comprehensive review of experimental, genetic, and clinical studies was conducted, focusing on the mechanisms of Aβ production, aggregation, and clearance, as well as the modulatory effects of ApoE isoforms. In vivo and in vitro data were analyzed to assess the interaction between Aβ and ApoE in AD models and human subjects, with attention to comparative studies across ApoE2, ApoE3, and ApoE4 carriers.

**Result:**

Findings indicate that Aβ aggregation and plaque deposition are significantly accelerated in the presence of ApoE4, which also impairs Aβ clearance and exacerbates neuroinflammation. ApoE2 appears to have a protective role, while ApoE3 demonstrates intermediate effects. Additionally, the interaction between Aβ and ApoE influences tau pathology, synaptic loss, and blood‐brain barrier integrity. These effects are mirrored in related disorders such as cerebral amyloid angiopathy and Lewy body dementia, highlighting a broader spectrum of ApoE‐mediated vulnerability.

**Conclusion:**

Amyloid‐beta and ApoE play central and interconnected roles in the pathogenesis of Alzheimer’s disease, influencing disease onset, progression, and response to therapy. The ApoE4 allele significantly amplifies Aβ‐driven pathology, suggesting its potential as both a prognostic marker and therapeutic target. Understanding these mechanisms provides critical insights into not only AD but also related neurodegenerative disorders with overlapping features, supporting a unified framework for future research and clinical intervention.